# ***In Vitro*** and ***In Vivo*** Modeling of Hydroxypropyl Methylcellulose (HPMC) Matrix Tablet Erosion Under Fasting and Postprandial Status

**DOI:** 10.1007/s11095-017-2113-7

**Published:** 2017-02-02

**Authors:** Benjamin Guiastrennec, Erik Söderlind, Sara Richardson, Alexandra Peric, Martin Bergstrand

**Affiliations:** 1 Pharmacometrics Group, Department of Pharmaceutical Biosciences, Uppsala University, Box 591, 75124 Uppsala, Sweden; 2 Pharmaceutical Technology and Development, AstraZeneca, Gothenburg, Sweden; 3 Janssen Pharmaceutica NV, Turnhoutseweg 30, B-2340 Beerse, Belgium; 4 Advanced Drug Delivery, Pharmaceutical Sciences, Innovative Medicines and Early Development, AstraZeneca, Gothenburg, Sweden; 5 Drug Metabolism and Pharmacokinetics, Cardiovascular and Metabolic Diseases, Innovative Medicines and Early Development, AstraZeneca, Gothenburg, Sweden

**Keywords:** food effect, hydroxypropyl methylcellulose, *in vitro in vivo* correlation, magnetic marker monitoring, NONMEM

## Abstract

**Purpose:**

To develop a model linking *in vitro* and *in vivo* erosion of extended release tablets under fasting and postprandial status.

**Methods:**

A nonlinear mixed-effects model was developed from the *in vitro* erosion profiles of four hydroxypropyl methylcellulose (HPMC) matrix tablets studied under a range of experimental conditions. The model was used to predict *in vivo* erosion of the HPMC matrix tablets in different locations of the gastrointestinal tract, determined by magnetic marker monitoring. In each gastrointestinal segment the pH was set to physiological values and mechanical stress was estimated in USP2 apparatus rotation speed equivalent.

**Results:**

Erosion was best described by a Michaelis–Menten type model. The maximal HPMC release rate (V_MAX_) was affected by pH, mechanical stress, HPMC and calcium hydrogen phosphate content. The amount of HPMC left at which the release rate is half of V_MAX_ depended on pH and calcium hydrogen phosphate. Mechanical stress was estimated for stomach (39.5 rpm), proximal (93.3 rpm) and distal (31.1 rpm) small intestine and colon (9.99 rpm).

**Conclusions:**

The *in silico* model accurately predicted the erosion profiles of HPMC matrix tablets under fasting and postprandial status and can be used to facilitate future development of extended release tablets.

**Electronic supplementary material:**

The online version of this article (doi:10.1007/s11095-017-2113-7) contains supplementary material, which is available to authorized users.

## Introduction

Extended release (ER) dosage forms are widely used to optimize drug concentration in plasma and improve patient compliance. Their main application lies in the reduction of the dosing frequency for drugs with short elimination half-life and in the reduction of side effects occurring for drugs exhibiting highly variable plasma concentrations. A common ER dosage form is the hydrophilic matrix tablet where an active pharmaceutical ingredient (API) is mixed with a hydrophilic polymer. Hydroxypropyl methylcellulose (HPMC) is frequently used to create hydrophilic matrices due to a safe and biodegradable profile, the compatibility with numerous drugs and the high drug load capacity. Upon ingestion of an ER tablet, water will diffuse into its core, causing the HPMC matrix to swell and form a rubbery gel. Hydrophilic API are released by diffusion into the dissolution media, while less soluble API as well as the HPMC polymer itself are being released by progressive erosion of the gel layer (1–4). The molecular weight and proportion of HPMC within an ER tablet can be modified to optimize the release rate of an API to a specific therapeutic need.

*In vitro* dissolution testing is a cornerstone in the development of solid dosage forms. These tests are designed and used for product quality control, but also to predict *in vivo* erosion time profiles by identifying the influential factors (*i.e.* covariates) of drug release. In the case of HPMC matrix tablets numerous influential factors linked to the characteristics of the polymer (*e.g.* molecular weight, degree of substitution), the formulation and the API (*e.g.* solubility) have been reported (3,5). *In vitro* testing experiments such as the United States pharmacopeia dissolution apparatus 2 (USP2) are typically conducted under a range of static experimental conditions (*i.e.* constant pH and mechanical stress) to characterize the effect of covariates on the release rate, but disregard the dynamic changes encountered *in vivo* along the gastrointestinal (GI) tract and between prandial statuses (6).

Magnetic marker monitoring (MMM) is a non-invasive, high resolution technique allowing the measurement in real-time of the erosion and the GI location of solid dosage forms. MMM relies on the measurement of the magnetic dipole generated by a magnetically labeled dosage form (7–9). More recently the MMM technique has been used to predict the *in vivo* GI transit of solid dosage form under different prandial statuses using *in silico* models (8).

The success of developing an ER tablet formulation with appropriate *in vivo* release characteristics relies on the ability to accurately quantify the effect of influential factors of the *in vivo* drug release, these factors include: tablet formulation, conditions throughout the GI tract and effect of prandial status. *In silico* models have been widely applied to *in vitro* predictions, yet only a few of these models have been utilized to make *in vivo* predictions of erosion time profiles (10–12). Hence, the present study aimed to link *in vitro* dissolution experiment data to *in vivo* erosion of ER tablets and to evaluate the effect of prandial status. For this purpose an *in silico* approach was used to 1) characterize the effect of formulation and experimental conditions based on *in vitro* erosion time profiles of four HPMC matrix tablets, 2) predict the *in vivo* erosion time profiles from a previously published MMM study under fasting and postprandial status (13).

## Materials and Methods

### Tablet Formulations

Four ER hydrophilic matrix tablet formulations were tested (Table I). The formulations no. 1 to 3 contained a fixed amount of total HPMC (40%) but variable proportions of high (303 kDa, Methocel K4M Premium; DowWolf, USA) and low (128 kDa, Methocel K100LV Premium; DowWolf, USA) molecular weight HPMC. Formulation no. 4 contained a reduced amount of HPMC (20%) and only used the low molecular weight HPMC. Calcium hydrogen phosphate (DCP) (DI-TAB®; Innophos, USA) was used as filler in all formulations to reach a total weight of about 350 mg. All tablets also contained fixed amounts of sodium stearyl fumarate (1%) (Pruv®; Moehs, Spain) and black iron oxide (1.4%) (Sicovit Schwarz 80E172, BASF), the latter being used as a label for the MMM.

**Table I:** Hydrophilic Matrix Tablets’ Formulation (13)

Component	Formulation			
	No. 1	No. 2	No. 3	No. 4
Methocel K4M % (*w*/*w*)	23.0	10.0	–	–
Methocel K100LV % (*w*/*w*)	17.0	30.0	40.0	20.0
Calcium hydrogen phosphate % (*w*/*w*)	57.6	57.6	57.6	77.6
Black iron oxide % (*w*/*w*)	1.4	1.4	1.4	1.4
Sodium stearyl fumarate % (*w*/*w*)	1.0	1.0	1.0	1.0

### *In Vitro* Experiments

The erosion properties of each formulation were evaluated *in vitro* using an USP2 (Hanson SR8 Plus Dissolution Test Station, USA) equipped with a stationary basket (6). The erosion was investigated under a range of experimental conditions: mechanical stress (*i.e.* rotation speed), pH and ionic strength (Table II). Biorelevant media were selected to mimic gastric (pH 1.2 and 232 mOsm/kg ionic strength) and small intestine (pH 6.8 and 98 mOsm/kg ionic strength) fluids. A third medium (pH 6.8 and 232 mOsm/kg ionic strength) was selected to differentiate the effects of pH and ionic strength on the release of HPMC. Each experimental setting was carried out as triplicate to ensure the reproducibility of the assay. In total, the *in vitro* data included 24 previously-published and 45 newly-generated tablet erosion profiles (Table II) (13).

**Table II:** Summary of *In Vitro* USP2 Apparatus Experimental Conditions

	pH 1.2		pH 6.8	
	Formulation	Mechanical stress (rpm)	Formulation	Mechanical stress (rpm)
Low ionic strength (*98 mOsm/kg*)	no. 1	–	no. 1	50^a^/100/150
	no. 2	–	no. 2	25/50^a^
	no. 3	–	no. 3	25/50^a^/100/150
	no. 4	–	no. 4	50^a^/100
High ionic strength (*232 mOsm/kg*)	no. 1	25/50^a^/150	no. 1	50
	no. 2	50^a^/100	no. 2	50
	no. 3	25/50^a^/150	no. 3	50
	no. 4	50^a^	no. 4	–

^a^Experiments part of a previous publication (13)

**Fig. 1:**
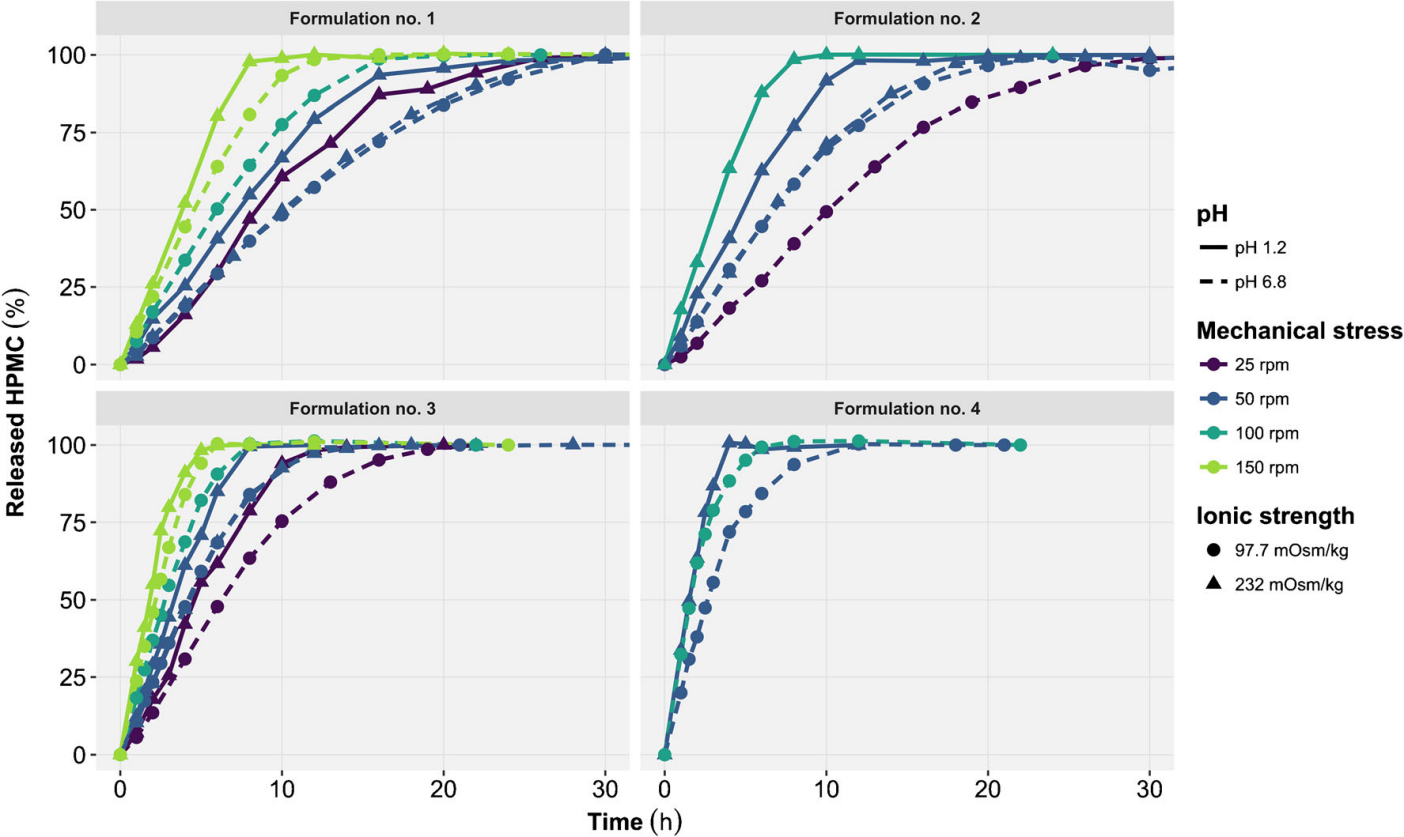
Released hydroxypropyl methylcellulose (HPMC) time profiles generated by the *in vitro* USP2 apparatus experiments. The profiles are represented as function of formulation (*panel*), pH (*line type*), mechanical stress (*color*) and ionic strength (*symbol*). Each point represents the mean of triplicate experiments.

Throughout this analysis HPMC release was used as surrogate for tablet erosion (Fig. 1). The concentration of HPMC in the release medium was determined by size exclusion chromatography with dual multi-angle light scattering and refractive index detection (SEC-MALS/RI) (13). A model published by *Jain et al.* was used to correlate the *in vitro* release of HPMC from the tablet to the decrease of the magnetic moment which the MMM method relies upon (13).

### Clinical Study

*In vivo* tablet erosion data were obtained from five healthy, young (range 27–34 years) male volunteers involved in a single center, open-label study (13). The study was conducted over six occasions separated by wash-out periods of at least 24 h following the last MMM measurement. Formulations no. 1 and 2 were administered under fasting status and formulations no. 3 and 4 under both fasting and postprandial status. Magnetized tablets were administered along with 240 mL of water at room temperature after an overnight fast of at least 10 h (fasting status) or 30 min after ingestion of a standardized high fat meal (postprandial status). MMM measurements of 10 min were initiated at the time of ingestion and conducted at intervals of 30 min to monitor tablet location in the GI tract and quantify the erosion. The GI tablet location was reported as three distinct regions: stomach, small intestine and colon. Volunteers were given standardized meal-break for lunch (+4 h), afternoon snack (+6 h) and dinner (+10 h). During these breaks no MMM measurements were conducted and volunteers were encouraged to walk or sit. Fluid intakes were also standardized from the time of tablet ingestion and up to 10 h afterward. MMM measurements were carried out until the net magnetization of the tablet was below 15% of its initial value (~60% HPMC released) but no longer than 14 h post administration.

All volunteers signed a written informed consent form. The study was performed in accordance with the ethical standards of the 1964 Helsinki declaration and its later amendments and was approved by the ethics committee of the medical chamber of Thüringen (Ethikkommission der Landesärztekammer Thüringen).

### Model Building

#### Overview of the Analysis

The present analysis has been conducted in three sequential steps. First, an *in silico* model was developed using the *in vitro* HPMC release time profiles to characterize the effect of influential factors (*i.e.* tablet formulation, experimental conditions). Secondly, the *in silico* model was applied to the prediction of *in vivo* HPMC release time profiles by using GI location (MMM data) to dynamically mimic the tablet environment properties throughout the GI. Lastly, the *in silico* model was used to simulate the *in vivo* HPMC release time profile for different formulation and prandial status combinations.

#### Software

The data analysis was performed using a nonlinear mixed-effects modeling approach implemented in the NONMEM software (v.7.3) and aided by functionalities of the PsN toolkit (v.4.4.2) (14). The first-order conditional estimation method with interaction (FOCE-I) and the ADVAN13 subroutine were used for parameter estimation. Graphical analysis and data management were conducted in R (v.3.2.0).

#### Model Selection and Diagnostics

Model selection was based on differences in the objective function value (OFV) using a significance level of *p* < 0.05 for nested models and on a comparison of the Akaike information criterion (AIC) for non-nested models. Graphical diagnostics, scientific plausibility and parameter uncertainty were also considered in the model evaluation. Parameter uncertainty reported as relative standard error (RSE) was obtained from the NONMEM sandwich estimator. Values missing at random, due to poor sample quality in the *in vitro* data (17%), were excluded from the analysis. No missing data were reported in the *in vivo* dataset.

Visual predictive checks (VPC) were used throughout model development to evaluate the predictive performance of the *in vitro* and *in vivo* models. This evaluation is done graphically by comparing the 5th, 50th and 95th percentiles of the observed data to the 95% confidence interval around the 5th, 50th and 95th percentiles of the simulated (*n* = 1000) data, grouped together within bins of the independent variable (*i.e.* time) (15). Prediction-corrected VPC were used to evaluate model performance across all experimental conditions and formulations within a single graphic. With prediction-corrected VPC the observed and simulated values of the dependent variable (*i.e.* released HPMC) are for each bin normalized to the typical model prediction (*i.e.* no parameter variability) at the median of the independent variable in the bin (15).

As per study protocol, no additional MMM measurements were conducted once the net magnetization of the ER tablet was below 15% of its initial value. To properly evaluate model performance, VPC with censoring were used in the diagnostic of the *in vivo* model by mimicking the study protocol. Censored VPC allowed the simulation of HPMC release profiles to be interrupted when the stopping criteria (*i.e.* low magnetization of the tablet) was met (15,16).

#### *In vitro* tablet erosion model

The *in silico* tablet erosion model was developed to characterize the *in vitro* HPMC release profiles. The predictive performance of a published mechanism-based model describing the HPMC release rate as function of the tablet surface area (Eq. 1) (10) was compared to a Michaelis-Menten type model (Eq. 2).

1 $$ \frac{dHPM{C}_{tablet}}{dt}=- R\cdot \left(\frac{HPM{C}_{dose}}{Tablet\; weight}\right)\cdot {\left(\frac{Tablet\; weight\cdot HPM{C}_{tablet}}{HPM{C}_{dose}}\right)}^{\gamma} $$

where HPMC_dose_ is the total amount of HPMC initially present in the tablet, HPMC_tablet_ the total amount of HPMC remaining in the tablet at a given time, R the basal HPMC release rate, and γ the power factor.

2 $$ \frac{dHPM{C}_{tablet}}{dt}=-\frac{V_{M AX}\cdot HPM{C}_{tablet}}{K_M+ HPM{C}_{tablet}} $$

where V_MAX_ is the maximal HPMC release rate and K_M_ corresponds to HPMC_tablet_ for which the release rate is half of V_MAX_. Both candidate models were pre-selected for their potential ability to describe the changes in the tablet erosion rate as a function of HPMC_tablet_—the erosion rate starting at a high and fairly constant value before gradually decreasing over time (Fig. 1). The models were implemented using differential equations to facilitate the inclusion of time varying covariates effect (*e.g.* increase in pH following gastric emptying) on the release rate of HPMC.

Addition of a lag-time to the HPMC release rate was evaluated to account for the presence of potential onset of HPMC release which may arise from the initial tablet hydration (17). The effect of experimental conditions (mechanical stress, pH and ionic strength) and tablet formulations (proportion of high molecular weight HPMC and DCP) were tested on the structural model parameters using a stepwise covariate modeling (SCM) approach as implemented in the PsN toolkit (14). In the forward inclusion step of the SCM, each covariate was tested in a univariate manner on each model parameter—including R and γ for the mechanism-based model and V_MAX_ and K_M_ for the Michaelis-Menten model. The most significant covariate-parameter relationship was then included in the model and taken forward. This process was performed in an iterative manner using the model from the previous step as starting point and testing the remaining covariate-parameter relationship until no more significant (*p* < 0.05) relationship was identified. In the backward deletion step of the SCM, the included covariates were removed one by one to more strictly (*p* ≥ 0.01) evaluate the statistical significance and omit potential redundant covariate-parameter relationship. All covariates were first tested by the SCM using relative linear effects as illustrated in Eq. 3:

3 $$ P R M= T{V}_{PRM}\cdot \left(1+{S}_{COV}\cdot \left( COV- CO{V}_{MED}\right)\right) $$

where TV_PRM_ is the estimated typical value of the model parameter (PRM), S_COV_ is effect slope of the covariate (COV) on TV_PRM_ and COV_MED_ is the median value of COV. The effect of mechanical stress and of the amount of high molecular weight HPMC were tested *in vitro* under a wider range of values enabling the evaluation of non-linearity in the covariate-parameter relationship (Table II). The non-linear relationships were evaluated using power (Eq. 4) and saturable Michaelis-Menten (Eq. 5) covariate-parameter relationships upon the linear covariate inclusion by the SCM.

4 $$ P R M= T{V}_{PRM}\cdot {\left(\frac{ CO V}{CO{V}_{MED}}\right)}^{PWR} $$

5 $$ P R M= T{V}_{PRM}\cdot \left(1+\frac{E_{MAX}\cdot COV}{CO{V}_{50}+ COV}\right) $$

where PWR is the power coefficient, E_MAX_ the maximal saturable effect of COV (bound between 0 and −1 in the case of an inhibitory effect) on the TV_PRM_ of PRM and COV_50_ the COV value leading half of E_MAX_. The between tablet variability (BTV) was investigated on model parameters and was assumed to follow a log-normal distribution. Proportional, additive and combined models were evaluated to describe the residual unexplained variability (*i.e.* measurement error, model misspecifications).

#### *In Vivo* Tablet Erosion Model

The selected *in vitro* model was used as a starting point to predict the *in vivo* HPMC release profiles data. Several modifications were carried out on the *in vitro* model to allow for proper description of *in vivo* data.

**Fig. 2:**
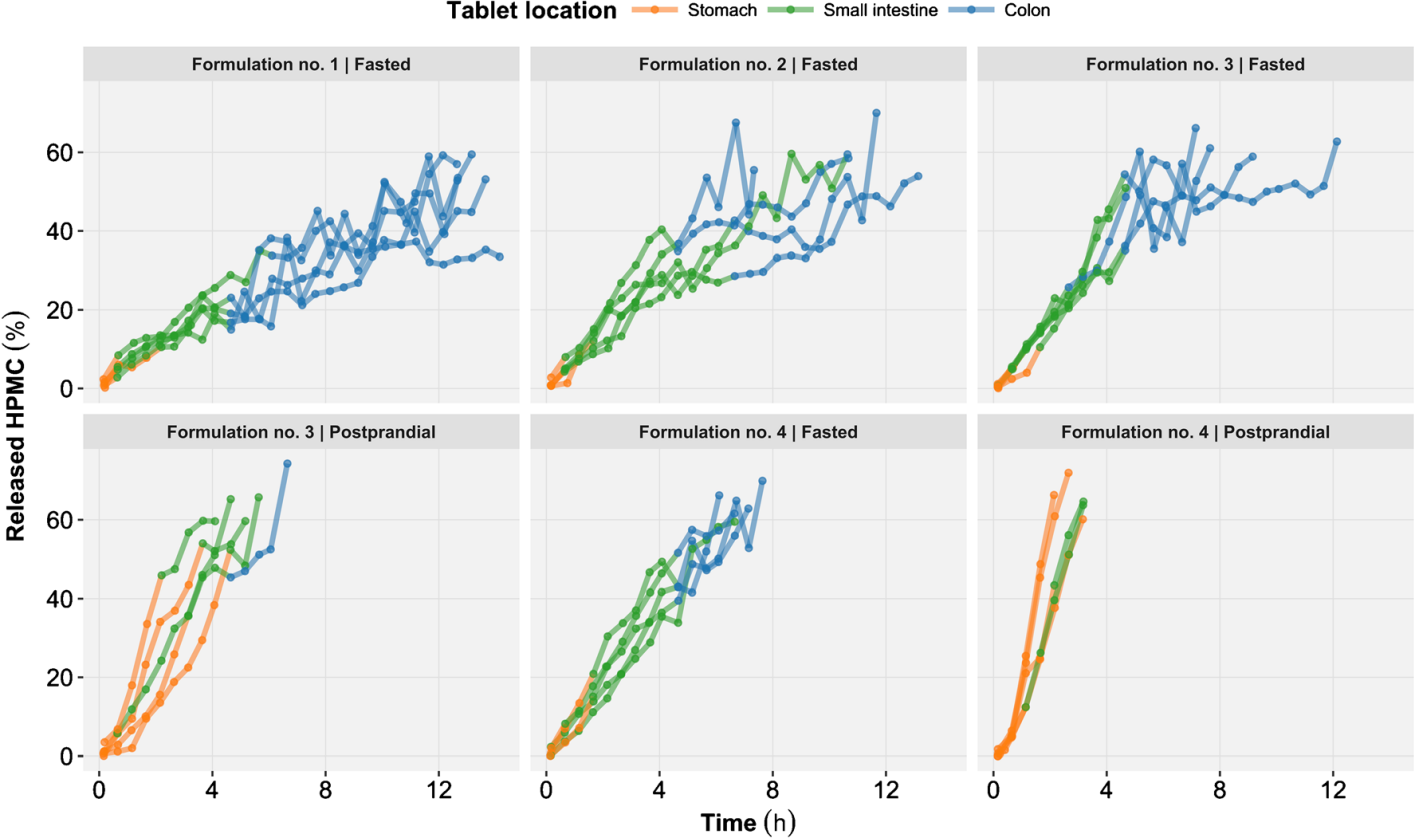
Released hydroxypropyl methylcellulose (HPMC) time profiles generated by the *in vivo* clinical study. The profiles are represented as function of formulation and prandial status (*panel*) as well as the tablet location in the gastrointestinal tract (*color*).

First, the typical values of all structural model parameters and the covariate effects were fixed to the *in vitro* model estimates (*i.e.* not re-estimated). Secondly, the variability in the model parameters was modified to account for the different layers of variability of the *in vivo* data, namely: the between subject variability (BSV) and between occasions variability (BOV) arising from the crossover design[34]. Thirdly, the residual unexplained variability structure was re-evaluated to account for the measurement error of the MMM. Fourthly, the GI tract tablet location (*i.e.* stomach, small intestine and colon) obtained from the MMM data was used to dynamically adjust the local pH and mechanical stress in the model. Graphical exploration of the data revealed consistent changes across individuals in the HPMC release rate throughout the small intestine (Fig. 2). To provide the model with the flexibility to account for these changes, three alternative approaches were tested to segment the small intestine into its proximal and distal parts. In the first approach, the individual small intestinal transit time (SITT) from the data along with the fractional length of proximal (45%) and distal (55%) small intestine were used to compute the time of transfer between the two segments (18). In the second approach the time of transfer between the proximal and distal small intestine for the population was estimated on relation to the time of gastric emptying. In the third approach it was fixed to one hour after the gastric emptying, as reported by *Bergstrand et al.* in a similar study (10). The resulting GI location (*i.e.* stomach, proximal small intestine, distal small intestine and colon) was used in the model to dynamically adjust the properties of the tablet environment. Literature values (mean and standard deviation) obtained from a similar population (Table III) were used as prior information to generate a distribution of pH within each GI location. The extent of mechanical stress on the tablet was estimated and expressed as USP2 apparatus rotation speed unit equivalent (rpm). Finally, for formulation no. 3 and 4, a postprandial effect was implemented by adjusting the distribution of pH values in the stomach, while the postprandial effect in other GI segments was assumed to be negligible (Table III). A potential postprandial effect was also investigated on the stomach mechanical stress.

**Table III:** pH Value in the Different Gastrointestinal Segments as a Function of the Prandial Status [35, 36]

Gastro-intestinal segment	Prandial status	pH value	
		Mean	SD
Stomach	Fasting ^a^	1.73	0.52
	Postprandial ^a^	4.90	0.81
Proximal small intestine	–	6.63	0.53
Distal small intestine	–	7.49	0.46
Colon	–	6.63	0.67

^a^Mean and standard deviation (SD) calculated from the median and interquartile range according to a method described by *Wan et al.* [37]

#### Simulations

Model simulations were performed to illustrate the expected *in vivo* HPMC release time profile with regard to formulation and prandial status. In order to generate a high number of new MMM GI location profiles, a first set of simulations were run from a previously published Markov model describing the GI transit of solid dosage form in fasting and postprandial status (8). The HPMC release profiles were then simulated by using the newly generated GI location profiles in the *in vivo* tablet erosion model. Finally, the simulations were summarized for each combination of formulation and prandial status by graphically representing the median and the 90% prediction interval of the simulated data.

## Results

### *In Vitro* Tablet Erosion Model

**Fig. 3:**
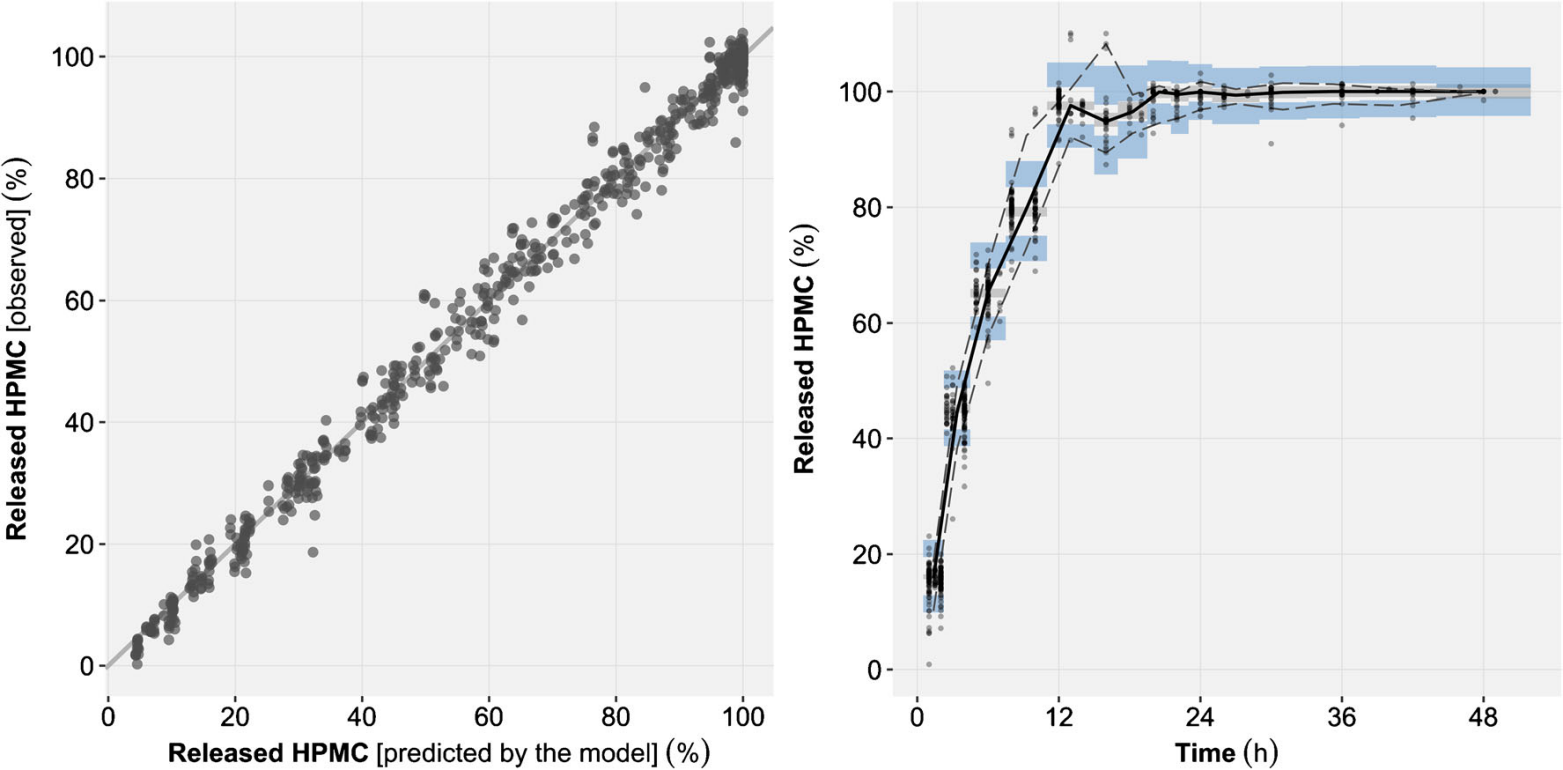
*Left:* goodness-of-fit plot of the *in vitro* model showing the correlation between the observed and the typical model predictions of released hydroxypropyl methylcellulose (HPMC) for all formulations and experimental conditions. *Right:* Prediction corrected visual predictive checks (VPC) of *in vitro* released HPMC time course for all formulations and experimental conditions. With prediction corrected VPC the observed and simulated values of the dependent variable (released HPMC) are for each bin normalized to the typical model prediction (*i.e.* no between tablet variability) at the median of the independent variable (*time*) in the bin. The median (*bold line*), 5th and 95th percentiles (*dashed lines*) of the observed data are compared to the 95% confidence intervals (*shaded areas*) for the median (*grey*), the 5th and 95th percentiles of the simulated (*n* = 1000) data (*blue*).

The Michaelis-Menten model (Eq. 2; AIC = 1959) displayed superior predictive performance than the mechanism-based model (Eq. 1; AIC = 2118). Moreover, the Michaelis-Menten model appropriately described the HPMC release rate of all four formulations under all studied experimental conditions and was thus selected as *in vitro* model (Fig. 3, Supplementary Materials S1).

The parameter estimates for the selected *in vitro* HPMC release model are reported in Table IV. Estimation of a lag-time (0.179 h) to account for the initial tablet hydration resulted in a significant model fit improvement. The BTV was estimated to a coefficient of variation (CV) of 5.96% for V_MAX_, but was no longer supported on K_M_ upon inclusion of the covariate effects and therefore not retained in the selected model. The SCM identified significant effects of pH, mechanical stress, the proportions of high molecular weight HPMC and DCP in the tablet on V_MAX_ (Eq. 6) and of pH was well of the proportion of DCP in the tablet on K_M_ (Eq. 7).

**Table IV:** Parameter Estimates for the *In Vitro* Tablet Erosion Model

Parameter (*units*)	Estimate (RSE%)
V_MAX_ (*mg*_*HPMC*_ */h*)	22.0 (2.5)
K_M_ (*mg*_*HPMC*_)	34.2 (6.1)
Lag-time (*h*)	0.179 (15)
MK4M_50-VMAX_ (*% w*/*w*)	18.7 (3.5)
S_DCP-KM_ (*% w*/*w* ^*−1*^) ^*a*^	0.0522 (33)
S_DCP-VMAX_ (*% w*/*w* ^*−1*^) ^*a*^	0.0332 (24)
S_pH-KM_ (*pH unit* ^*−1*^) ^*a*^	0.0935 (15)
S_pH-VMAX_ (*pH unit* ^*−1*^) ^*a*^	−0.0319 (24)
S_rpm-VMAX_ (*rpm* ^*−1*^) ^*a*^	0.0115 (2.9)
BTV V_MAX_ (*CV %*)	5.96 (17)
Additive error ^b^ (*mg*_*HPMC*_)	1.95 (14)

*BTV* between tablet variability, *CV* coefficient of variation, *DCP* calcium hydrogen phosphate, *K*_*M*_ hydroxypropyl methylcellulose (HPMC) amount left in tablet at which V_MAX_ is reduced by 50%, *MK4M*_*50-VMAX*_ amount of high molecular weight HPMC at which V_MAX_ is reduced by 50%, *RSE* relative standard error, *S*_*DCP-KM*_ DCP effect slope on K_M_, *S*_*DCP-VMAX*_ DCP effect slope on V_MAX_, *S*_*pH-KM*_ pH effect slope on K_M_, *S*_*pH-VMAX*_ pH effect slope on V_MAX_, *S*_*rpm-VMAX*_ mechanical stress effect slope on V_MAX_, *V*_*MAX*_ maximal HPMC release rate from the tablet

^a^covariate effects reported as relative change in the model parameter typical value for each unit change of the covariate in reference to its median

^b^reported on the standard deviation scale

**Fig. 4:**
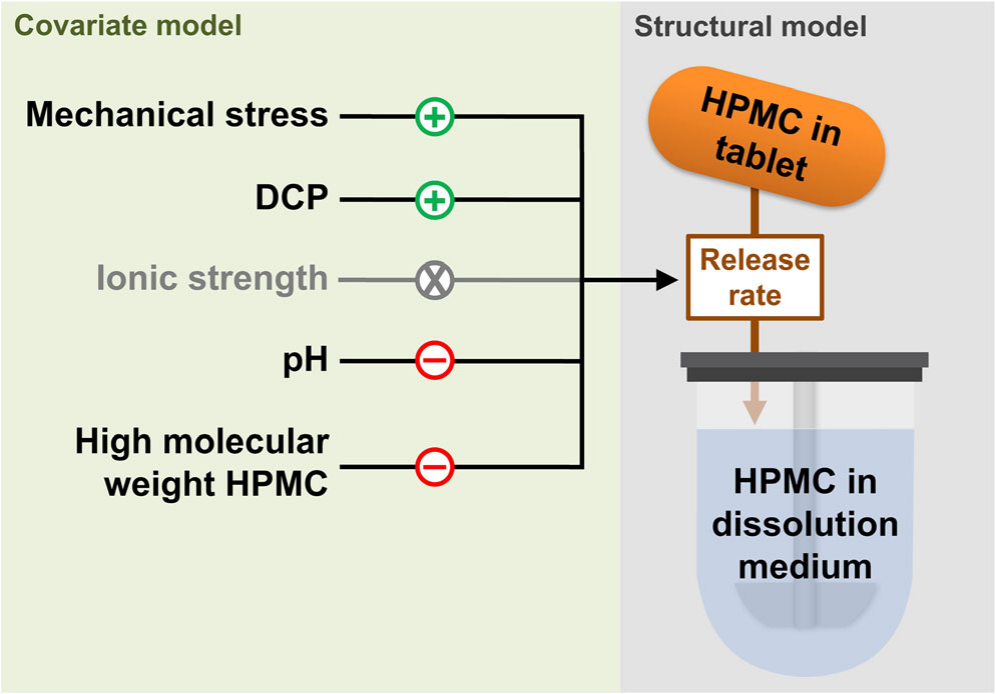
Schematic representation of the structural model (*right*) where hydroxypropyl methylcellulose (HPMC) is being released from the tablet into the dissolution medium. The effects of covariate (*left*) on the tablet erosion rate are also represented. The *green* (+) symbols represent a stimulation, the *red* (−) symbols an inhibition and the *grey* (x) symbol an absence of observable covariate effect on the tablet erosion rate. DCP: calcium hydrogen phosphate.

**Fig. 5:**
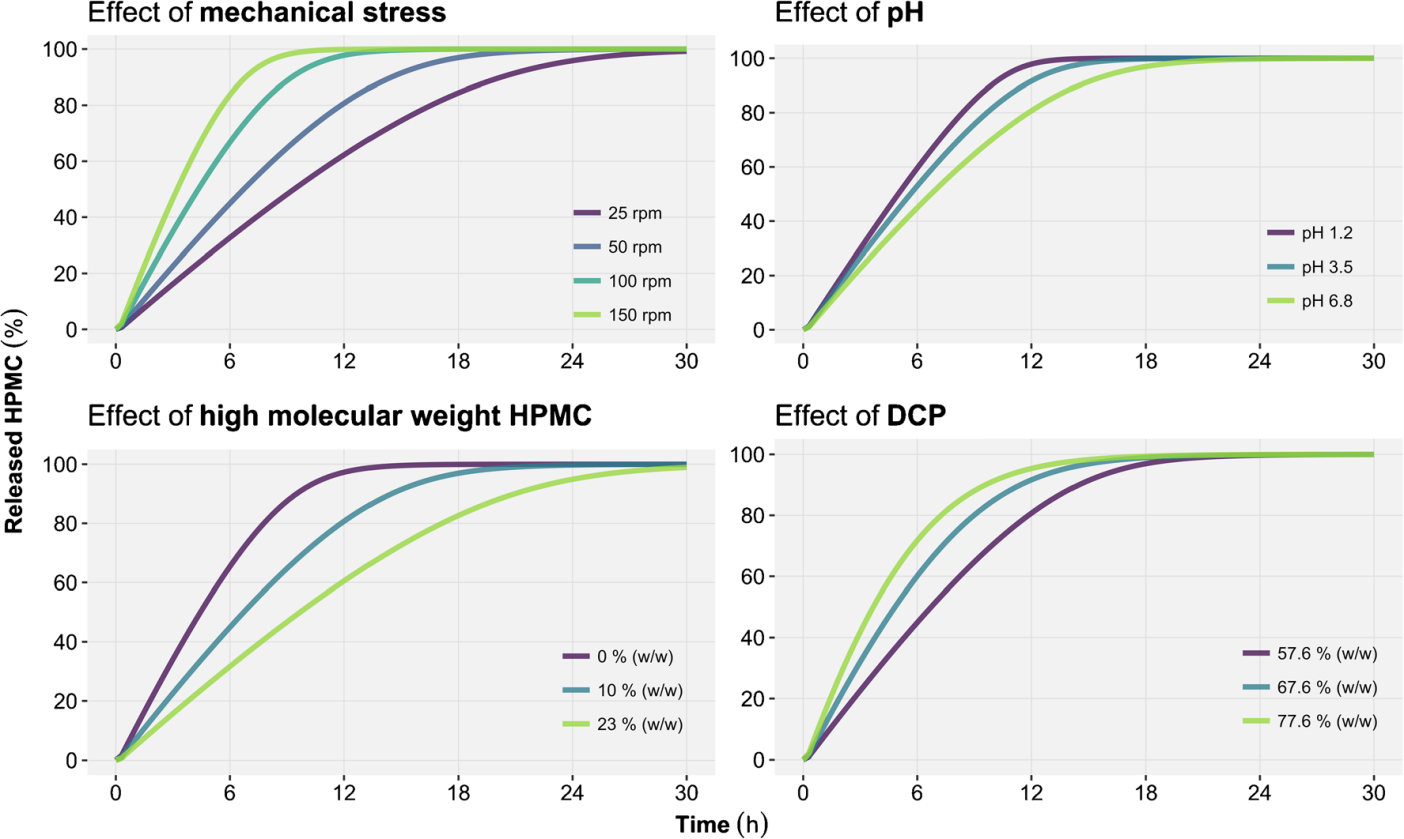
Illustration of the predicted *in vitro* effect of: mechanical stress (*top left*), pH (*top right*), high molecular weight hydroxypropyl methylcellulose (HPMC) (*bottom left*) and calcium hydrogen phosphate (DCP) (*bottom right*) on released HPMC time profiles.

6 $$ {V}_{MAX}= T{V}_{V MAX}\cdot {E}_{rpm- VMAX}\cdot {E}_{pH- VMAX}\cdot {E}_{DCP- VMAX}\cdot {E}_{MK4 M- VMAX}\cdot {e}^{\eta} $$

7 $$ {K}_M= T{V}_{K M}\cdot {E}_{pH- KM}\cdot {E}_{DCP- KM} $$

where TV_VMAX_ and TV_KM_ represent the typical (*i.e.* formulation no. 3, pH 6.8, mechanical stress = 50 rpm) parameter values of V_MAX_ and K_M_. E_rpm-VMAX_, E_pH-VMAX_, E_DCP-VMAX_ and E_MK4M-VMAX_ represent the relative effects of mechanical stress, pH, DCP and high molecular weight HPMC, respectively, on TV_VMAX_. The symbol η represents the BTV on V_MAX_. E_pH-KM_ and E_DCP-KM_ represent the relative effects of pH and DCP on K_M_. E_MK4M-VMAX_ was best described by a saturable relationship (Eq. 8) which resulted in a model fit improvement over a linear relationship.

8 $$ {E}_{M K4 M- VMAX}=1-\frac{ M K4 M}{ M K4{M}_{50- VMAX}+ MK4 M} $$

where MK4M is the proportion of high molecular weight HPMC and MK4M_50-VMAX_ the MK4M leading to a decrease by half of E_MK4M-VMAX_. In Eq. 8 E_MAX_ was not statistically different from −1 and hence was fixed to a full inhibitory effect in the model. No effect of the ionic strength could be detected on any of the model parameters. A schematic representation of the structural and covariate models is illustrated in Fig. 4. Moreover, a visual representation of the effect of significant covariate relationships on the released HPMC time profiles is provided in Fig. 5. Finally, the residual unexplained variability was best described by an additive error model.

### *In Vivo* Tablet Erosion Model

**Fig. 6:**
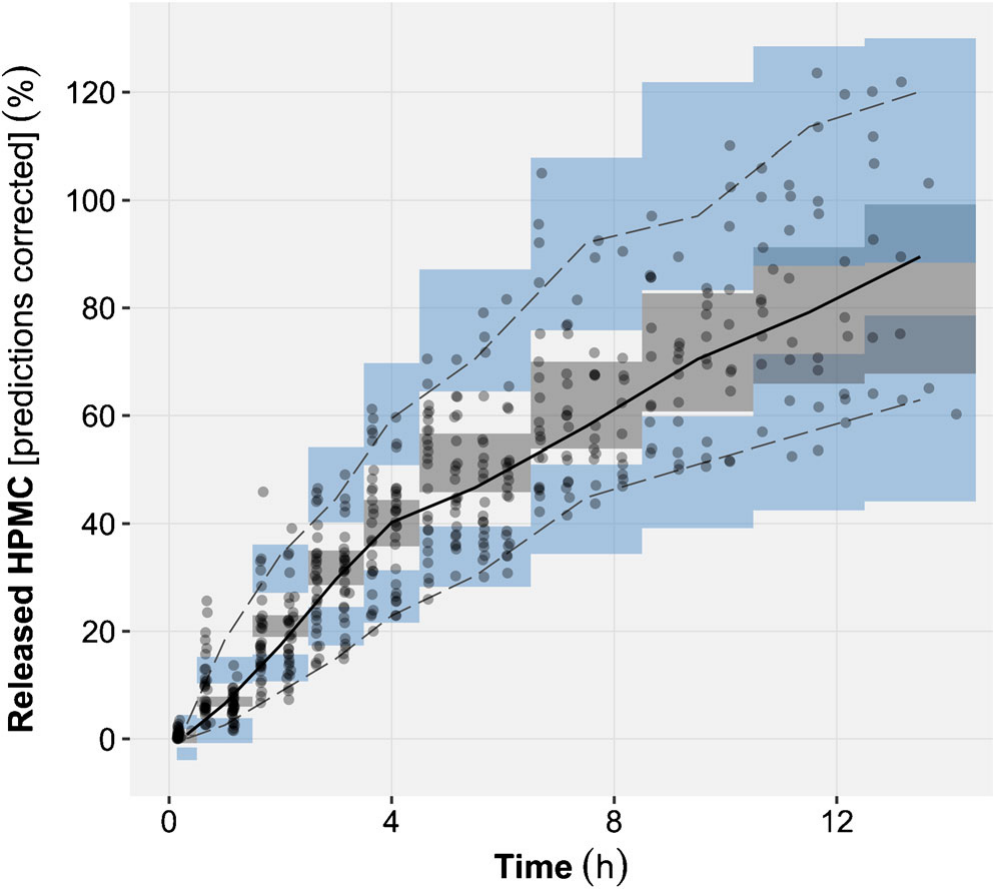
Prediction-corrected visual predictive check of the *in vivo* released hydroxypropyl methylcellulose (HPMC) time profiles for all formulations and prandial statuses. With prediction corrected VPC the observed and simulated values of the dependent variable (released HPMC) are for each bin normalized to the typical model prediction (*i.e.* no between tablet variability) at the median of the independent variable (*time*) in the bin. The median (*bold line*), 5th and 95th percentiles (*dashed lines*) of the observed data are compared to the 95% confidence intervals (*shaded areas*) for the median (*grey*), the 5th and 95th percentiles of the simulated (*n* = 1000) data (*blue*).

**Fig. 7:**
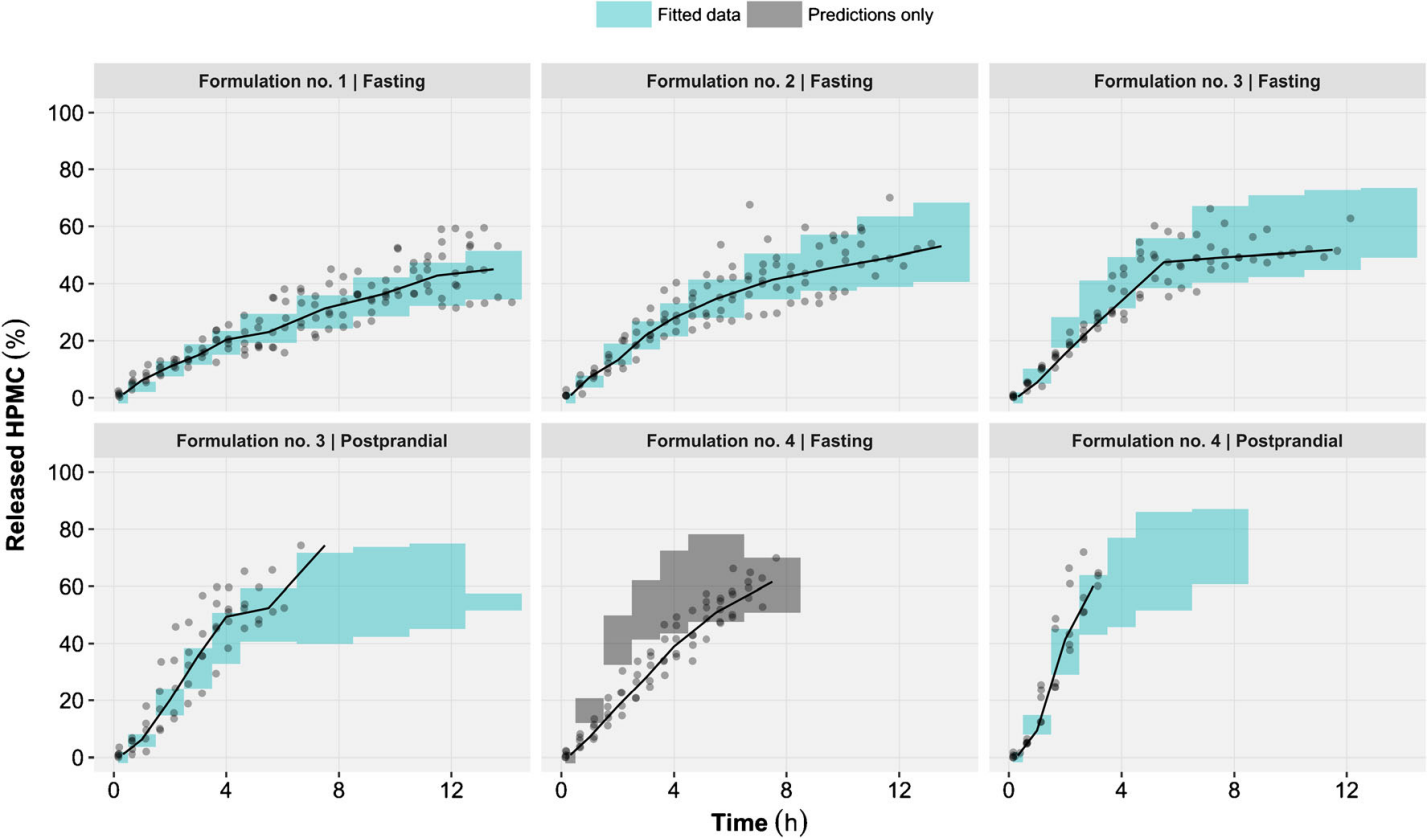
Visual predictive checks of the *in vivo* released hydroxypropyl methylcellulose (HPMC) time profiles, stratified by formulation and prandial status. The simulated released HPMC were censored once the net magnetization of a tablet was below 15% of its initial value. Censoring was utilized in the simulations as per study protocol where no subsequent measurements were performed once a threshold was met thus allowing to properly diagnose the model predictions. For each panel, the median (*black lines*) of the observed data (*grey dots*) is compared to the 95% confidence interval of the simulated (*n* = 1000) median (*shaded area*). The panels in *blue* represent the data used during the model development process, while the panel in grey represents the data where only predictions were made.

Predictions from the selected *in vivo* tablet erosion model were overall in good agreement with the observed released HPMC time profiles (Fig. 6). Under the fasting status, HPMC release from the formulation no. 4 (*i.e.* lowest HPMC content) were systematically over predicted by the model (Fig. 7). Thus, the data from formulation no. 4 under fasting status were excluded during the final parameters estimation, but were included for model diagnostics and simulations. This approach was selected to prevent the introduction of an estimation bias in the model parameters due to model misspecification. The parameter estimates for the selected *in vivo* tablet erosion model have been reported in Table V and the model code in the Supplementary Materials S2. The small intestine segmentation into its proximal and distal parts significantly improved model predictions. A model where the time of transfer between proximal and distal small intestine was fixed to one hour after gastric emptying gave the best results. The mechanical stress was estimated for stomach (39.5 rpm), proximal small intestine (93.3 rpm), distal small intestine (31.1 rpm) and colon (9.99 rpm). Re-estimation of the lag-time (0.410 h) significantly improved model predictions. The estimated BSV and BOV on V_MAX_ were 14.9 and 15.5% respectively. BSV was also tested on K_M_ but was not statistically significant and thus was not retained in the selected *in vivo* model. A postprandial effect was implemented on stomach pH, although the estimation of an additional effect on mechanical stress was not supported by the data. The residual unexplained variability was best described by a combined (*i.e.* additive and proportional) model.

**Table V:** Parameter Estimates for the *In Vivo* Tablet Erosion Model

Parameter (*units*)	Estimate (RSE%)
RPM_ST_ (*rpm*)	39.5 (24)
RPM_PSI_ (*rpm*)	93.3 (12)
RPM_DSI_ (*rpm*)	31.1 (21)
RPM_CO_ (*rpm*)	9.99 (54)
Lag-time (*h*)	0.410 (9.6)
BSV on V_MAX_ (*CV %*)	14.9 (23)
BOV on V_MAX_ (*CV %*)	15.5 (36)
Proportional error (*CV %*)	14.2 (13)
Additive error^a^ (*mg*_*HPMC*_)	1.86 (21)

*BOV* between occasion variability, *BSV* between subject variability, *CV* coefficient of variation, *HPMC* hydroxypropyl methylcellulose, *RPM*_*CO*_ mechanical stress in colon, *RPM*_*DSI*_ mechanical stress in distal small intestine, *RPM*_*PSI*_ mechanical stress in proximal small intestine, *RPM*_*ST*_ mechanical stress in stomach, *RSE* relative standard error

^a^reported on the standard deviation scale

## Discussion

The present study demonstrated how the data from *in vitro* dissolution experiment could be integrated *via* an *in silico* approach to accurately predict *in vivo* tablet erosion of ER tablets under fasting and postprandial conditions. The *in silico* model was developed using *in vitro* USP2 apparatus dissolution data and applied to the characterization of tablet formulations and experimental conditions effects on the HPMC matrix tablet erosion. The model was then used to the predict the *in vivo* tablet erosion profiles by integrating MMM tablet location data. The resulting *in silico* model could be applied to the prediction of the *in vivo* erosion for similar hydrophilic matrix formulations and to the refinement of the experimental conditions used *in vitro*.

Multiple *in vitro* tablet erosion models with various levels of complexity are available in the literature (21–23). These models were not investigated in the present study since they could not easily be applied to the dynamic conditions (*e.g.* changes in pH and mechanical stress) encountered *in vivo* throughout the GI tract. The *in vitro* predictive performance of the mechanism-based model developed by *Bergstrand et al.* in a similar study (10) was compared to a Michaelis-Menten model. Both models appropriately predicted the *in vitro* erosion time profiles, however the Michaelis-Menten model (AIC = 1959) gave a better description of the data than the mechanism-based model (AIC = 2118). The mechanism based model assumes that the shape of a tablet remains constant throughout its disintegration, interestingly this assumption may have not been fully supported by the data.

The estimation of a lag-time of erosion (0.179 h) to describe the initial tablet hydration significantly improved the *in vitro* model predictions (17,24). A 1 h lag-time has also been reported *in vitro* by *Tajarobi et al.* with similar tablet formulation (24). Unlike the present study the published value was not estimated but obtained from a direct observation at the first time point of the experiment which is likely to explain the difference between them. The estimation of a more pronounced *in vivo* lag-time (0.410 h) is also in line with values reported by *Ghimire et al.* and is believed to arise from the higher viscosity of the fluids in the stomach (17).

The identified effects of tablet formulations and experimental conditions on the release rate of HPMC were all in line with reported covariates of HPMC matrix tablet erosion (1,3,17,24). The ratio of heavy to low molecular weight HPMC (formulation no. 1 to 3) was predicted to be inversely and non-linearly correlated to V_MAX_. Reduced water diffusion rate into the tablet has been reported with higher HPMC molecular weight (3,13,25). An increased fraction of DCP (formulation no. 4) was predicted to increase both V_MAX_ and K_M_. Previous studies have reported that formulation additives such as DCP or mannitol increase the diffusion of water into the tablet and thus the maximal erosion rate as compared to a pure HPMC matrix tablets (1,24). The predicted effect of pH on V_MAX_ and K_M_ is likely related to the presence of DCP, which solubility is altered by pH and hence affects the water entry into the tablet as described above (1). Mechanical stress had the strongest effect on V_MAX_ and displayed a linear relationship across the tested range (25–150 rpm). This effect depicts individual polymer chains being disjointed from the gel matrix under the action of the shear forces (24). Unlike findings from *Bergstrand et al.*, no significant effect of ionic strength was detected despite *in vitro* experiments conducted at 98 and 232 mOsm/kg with a pH of 6.8 (Table II) (10). The graphical overlay between the erosion profiles at low and high ionic strength (Fig. 1, formulations no. 1 and 2, mechanical stress = 50 rpm, pH 6.8) indicates that this finding is related to the *in vitro* experiment data rather than model misspecifications. Others have reported the influence of the HPMC quality and of the filling agent’s solubility on the sensibility of tablet erosion to ionic strength which could explain the differences with findings from *Bergstrand et al.* (26–28). Indeed, the formulations studied herein were mostly composed of poorly soluble DCP and used K-quality HPMC whereas, the formulations studied by *Bergstrand et al.* used a highly soluble API along with E-quality HPMC (10).

**Fig. 8:**
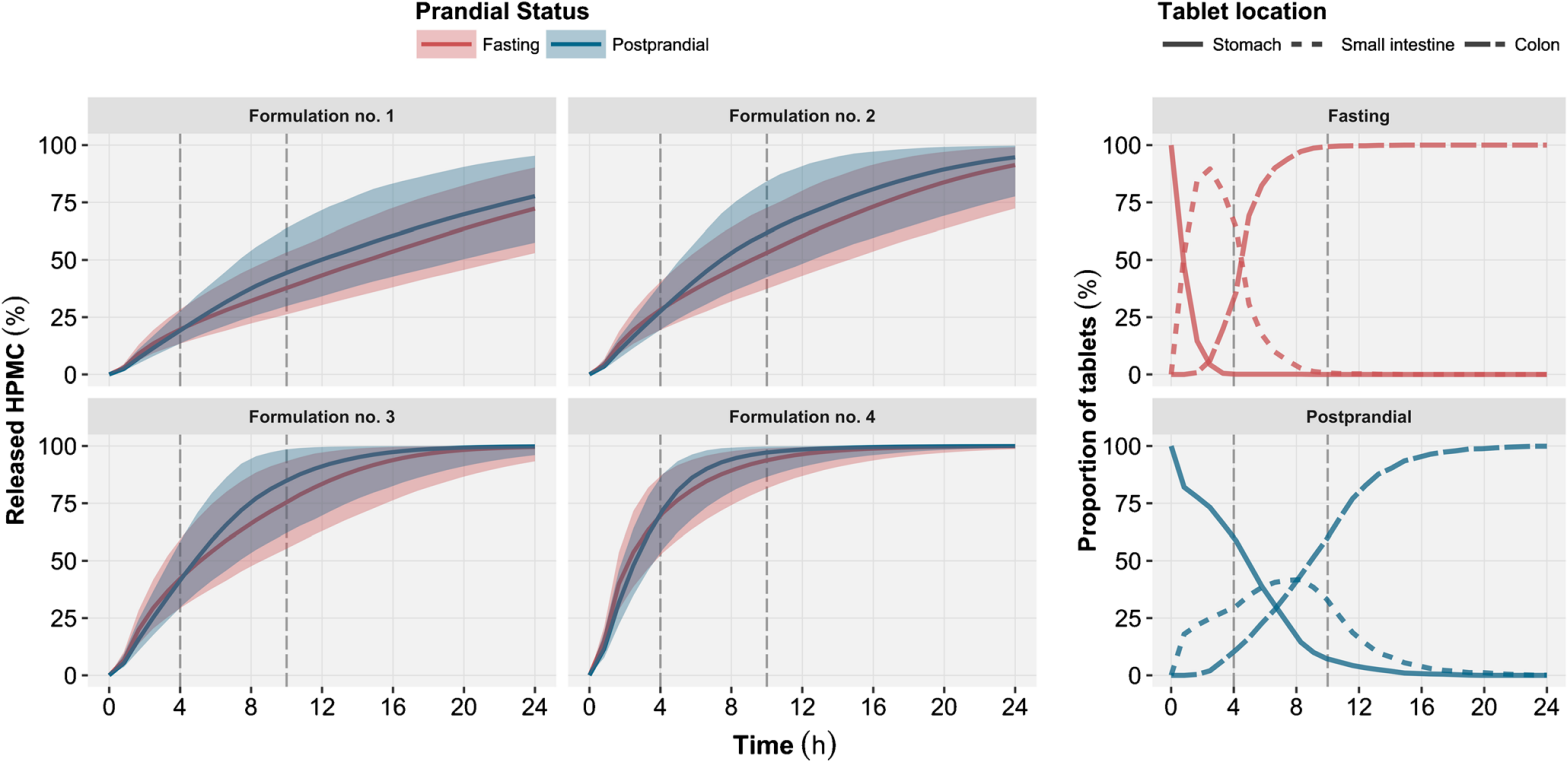
*Left:* Simulated *in vivo* released hydroxypropyl methylcellulose (HPMC) time profiles (*left*) as a function of prandial status (*color*) and formulation (*panel*). The vertical dashed lines represent the time of lunch (+4 h) and dinner (+10 h) intake for both the fasting and postprandial conditions. For each simulated prandial status and formulation, the median (*continuous line*) and the 90% prediction interval (*shaded area*) were computed from 1000 simulated released HPMC profiles. *Right:* Graphical representation of the proportion of simulated tablet location in each gastrointestinal segment (*line type*) as function of the prandial status (*color*). These tablet locations were used by the *in vivo* model to generate the released HPMC profiles.

The *in vivo* model predictions were overall in good agreement with the data. However, the predictions for formulation no. 4 under fasting status were unexpectedly poor (Fig. 7). This data was thus subsequently excluded from the final parameters estimation to the prevent introduction of a bias in the parameter estimates. Tablets with a HPMC content inferior to the percolation threshold (~30–35% *w*/*w*) such as formulation no. 4 (20% *w*/*w*) are however likely to exhibit poor *in vivo* behavior. With an HPMC content below the percolation threshold the formed gel layer becomes weaker and more prone to disruption by the shear forces (1,17). The predicted *in vivo* release of HPMC for formulation no. 4 (Fig. 8) should however be interpreted carefully.

Assuming homogenous pH and mechanical stress throughout the entire small intestine transit did not provide an optimal description of the data. Surprisingly, among the three tested small intestine segmentation approaches, the use of individual SITT did not perform well as opposed to a segmentation one hour post gastric emptying for all individuals. This finding could be due to a long tablet residence time in the ileocecal junction, whereas the transit time throughout the proximal small intestine might have been relatively constant (29).

The *in vivo* mechanical stress estimates were consistently lower in all GI segments than previously reported by *Bergstrand et al.* in a similar study with tablets containing an API (10). These discrepancies can potentially be explained by differences in the tablet formulation, in the present study tablets no API was used. In the *in vivo* model described by *Bergstrand et al.* significant effects of the ionic strength of the media and of the amount of API in the tablet were identified on the erosion rate. In addition, *Bergstrand et al.* tested two different values of intestinal ionic strength in the model which resulted in marginally different estimates of mechanical stress (10). As previously mentioned, the sensitivity of a formulation to ionic strength is function of the osmotic pressure generated by API and filling agent (24,26). In the case of DCP, the generated osmotic pressure is limited due to its low solubility (24). This may explain the absence of an ionic strength effect in the studied formulations and consequently the discrepancies with the previously reported mechanical stress estimates (10).

The parameter estimates for the mechanical stress in the proximal small intestine was estimated to 93.3 rpm before dropping to 31.1 rpm in the distal small intestine. The estimate for proximal small intestine was more than two times the estimate for the stomach (39.5 rpm). The high proximal intestine estimate could be explained by some disruption or damage of the tablet or the gel layer throughout the gastric emptying process (23,30). Additionally, while the estimate of mechanical stress in stomach mainly arose from the postprandial data due to the prolonged gastric residence, the estimates for small intestine and colon mainly arose from the fasting data. Hence, in the postprandial state tablets may be have been surrounded by meal components creating a viscous medium with low water content. Such effect of viscosity on the tablet erosion rate have been reported *in vivo* when tablets were ingested along with viscous meals (31,32). The same mechanism may also explain the very small estimate of the mechanical stress in colon (9.99 rpm) as water is extensively being reabsorbed in that part of the intestine, which gradually increases the viscosity of the surrounding medium.

In the present study, the prandial effect was implemented as a change in stomach pH, although in line with similar studies, no effect was observed on the mechanical stress (10,33). While the authors do not exclude the hypothesis of an increased mechanical stress in postprandial stomach this effect was not supported by the current data where the tablet erosion data was limited in fasting stomach due to a short residence time (Fig. 2). Despite this, the model properly described the differences between fasting and postprandial status for formulation no 3. (Fig. 7) through the increased residence time in stomach and the relatively high mechanical stress encountered in stomach as compared to the mechanical stress encountered in distal small intestine and colon.

The developed *in silico* model was used to perform simulations of different formulation and prandial status (Fig. 8). The model also offers the opportunity to simulate new scenarios (*e.g.* new formulations) while accounting for the effect of covariates and the between subject variability in GI transit and pH. To perform these simulations the *in silico* model was used along with a previously developed continuous Markov model describing the GI transit of solid dosage form under fasting and postprandial status (8). This implementation enables the simulation of new clinical trial design, which could be used to explore the HPMC release under different food patterns or with multiple tablet administration. Such simulations can be used to support of the development process by providing information on the expected typical tablet erosion profiles and the magnitude of variability. If used along with pharmacokinetic data, the model could be extended to predict the variability in the absorption profile of API released from ER formulations (19). It should be noted that the predictive performance of the proposed *in vivo* HPMC matrix tablet erosion model is appropriate for interpolation but has not been evaluated outside the studied range of covariates.

The present study is primarily devoted to ER tablets with an erosion-controlled drug release mechanism. The absence of API in the investigated tablets provided a fundamental understanding of the mechanisms of HPMC tablet erosion and the impact of covariates. Combined with API properties, this knowledge could enable to better predict *in vivo* behavior of API containing formulations. The drawback of this approach is that the *in vivo* absorption process of the studied formulations could not be evaluated. Furthermore, the proposed model relies on simplification of the shear forces and pH encountered *in vivo*. The MMM data used to determine the GI location of the tablets only reported three different locations, namely: stomach, small intestine and colon. In a similar study by *Bergrstrand et al.* more accurate MMM measurement allowed the distinction between proximal and distal stomach and the different sections of the colon which could have improved the current model predictions (10). The model also assumed a time-constant pH in the postprandial stomach, reports have however shown a return toward fasting values after several hours (20). Development of model describing the postprandial evolution of pH in stomach using data from frequent *in vivo* sampling could help to improve the current model predictions (20). The poor predictive performance of the model for formulation no. 4 under fasting status were unexpected. While it has been reported that tablets containing low amount of polymer are more likely to exhibit poor *in vivo* behavior, this may indicate that some factors in the *in vitro-in vivo* translation are yet to be characterized.

## Conclusion

This work illustrates how an *in silico* erosion model for HPMC matrix tablets was developed using a simple set of *in vitro* USP2 apparatus dissolution profiles to predict the *in vivo* erosion. The presented methodology described how the *in vitro* effects of tablet formulations and experimental conditions were integrated in the *in silico* model to predict erosion time profiles of multiple HPMC matrix tablets under fasting and postprandial status. This study also exemplified how the *in silico* model can be used to inform decision-making through simulation of a typical tablet erosion profile and the extent of its variability.

## Electronic supplementary material

Below is the link to the electronic supplementary material.

Fig. S1 (DOCX 1096 kb)

Fig. S2 (DOCX 46 kb)

## Abbreviations

γ: Power factor parameter
η: BTV of V_MAX_
AIC: Akaike information criterion
API: Active pharmaceutical ingredient
BOV: Between occasion variability
BSV: Between subject variability
BTV: Between tablet variability
COV: A covariate
COV_50_: COV value leading to half of the maximal effect
COV_MED_: Median value of COV
CV: Coefficient of variation
DCP: Calcium hydrogen phosphate
E_COV_: Relative change in TV_PRM_ for each unit change of COV in reference to COV_MED_
E_DCP-KM_: Relative effect of DCP on the typical value of K_M_
E_DCP-VMAX_: Relative effect of DCP on the typical value of V_MAX_
E_MAX_: Maximal saturable effect of COV on TV_PRM_
E_MK4M-VMAX_: Relative effect of MK4M on the typical value of V_MAX_
E_pH-KM_: Relative effect of pH on the typical value of K_M_
E_pH-VMAX_: Relative effect of pH on the typical value of V_MAX_
ER: Extended release
E_rpm-VMAX_: Relative effect of mechanical stress on the typical value of V_MAX_
FOCE-I: First-order conditional estimation method with interaction
GI: Gastrointestinal
HPMC: Hydroxypropyl methylcellulose
HPMC_dose_: Total amount of HPMC initially present in the tablet
HPMC_tablet_: Total amount of HPMC remaining in the tablet at a given time
K_M_: Amount of HPMC in tablet at which the release rate is half of V_MAX_
MK4M: Proportion of high molecular weight HPMC in the tablet
MK4M_50-VMAX_: MK4M leading to a decrease of 50% in the typical value of V_MAX_
MMM: Magnetic marker monitoring
OFV: Objective function value
PRM: A model parameter
PWR: Power coefficient parameter
R: Basal HPMC release rate parameter
RSE: Relative standard error
SCM: Stepwise covariate modeling
S_COV_: Effect slope of COV on TV_PRM_
SITT: Small intestinal transit time
TV_KM_: Typical value of the K_M_ parameter
TV_PRM_: Typical value of a parameter
TV_VMAX_: Typical value of the V_MAX_ parameter
USP2: United States pharmacopeia dissolution apparatus 2
V_MAX_: Maximal HPMC release rate
VPC: Visual predictive checks
