# Supplementary material for: In Vitro and In Vivo Modeling of Hydroxypropyl Methylcellulose (HPMC) Matrix Tablet Erosion Under Fasting and Postprandial Status
Source: Pharm Res. 2017 Feb 2;34(4):847–59. doi: 10.1007/s11095-017-2113-7 (PMC5336534; doi:10.1007/s11095-017-2113-7)
Supplement: Supplementary file 1 — (DOCX 1096 kb) [file 11095_2017_2113_MOESM1_ESM.docx]

**
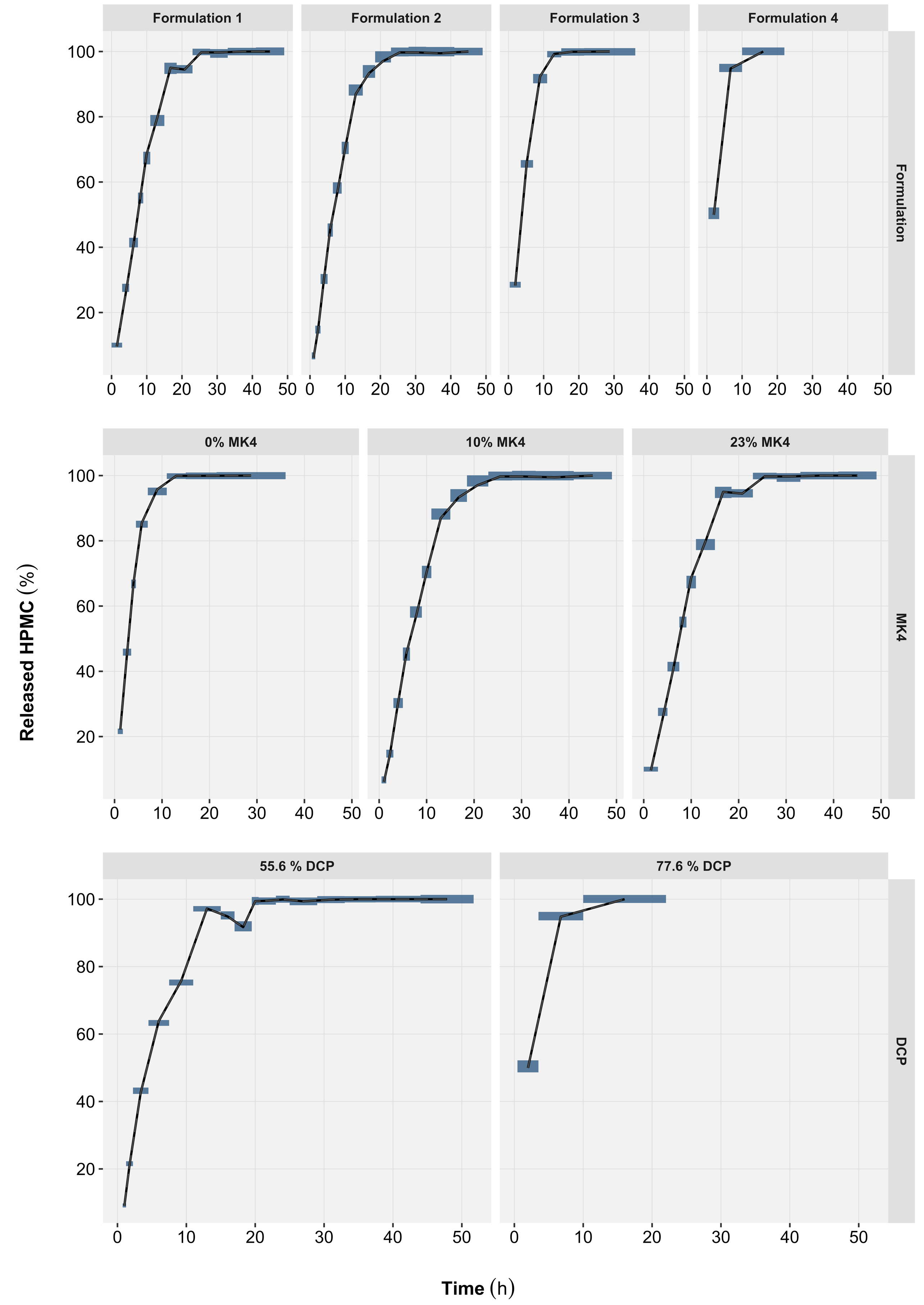
Figure S1a:** prediction corrected VPC of the formulation related covariates

**Figure S1b:** prediction corrected VPC of the experimental conditions related covariates

**
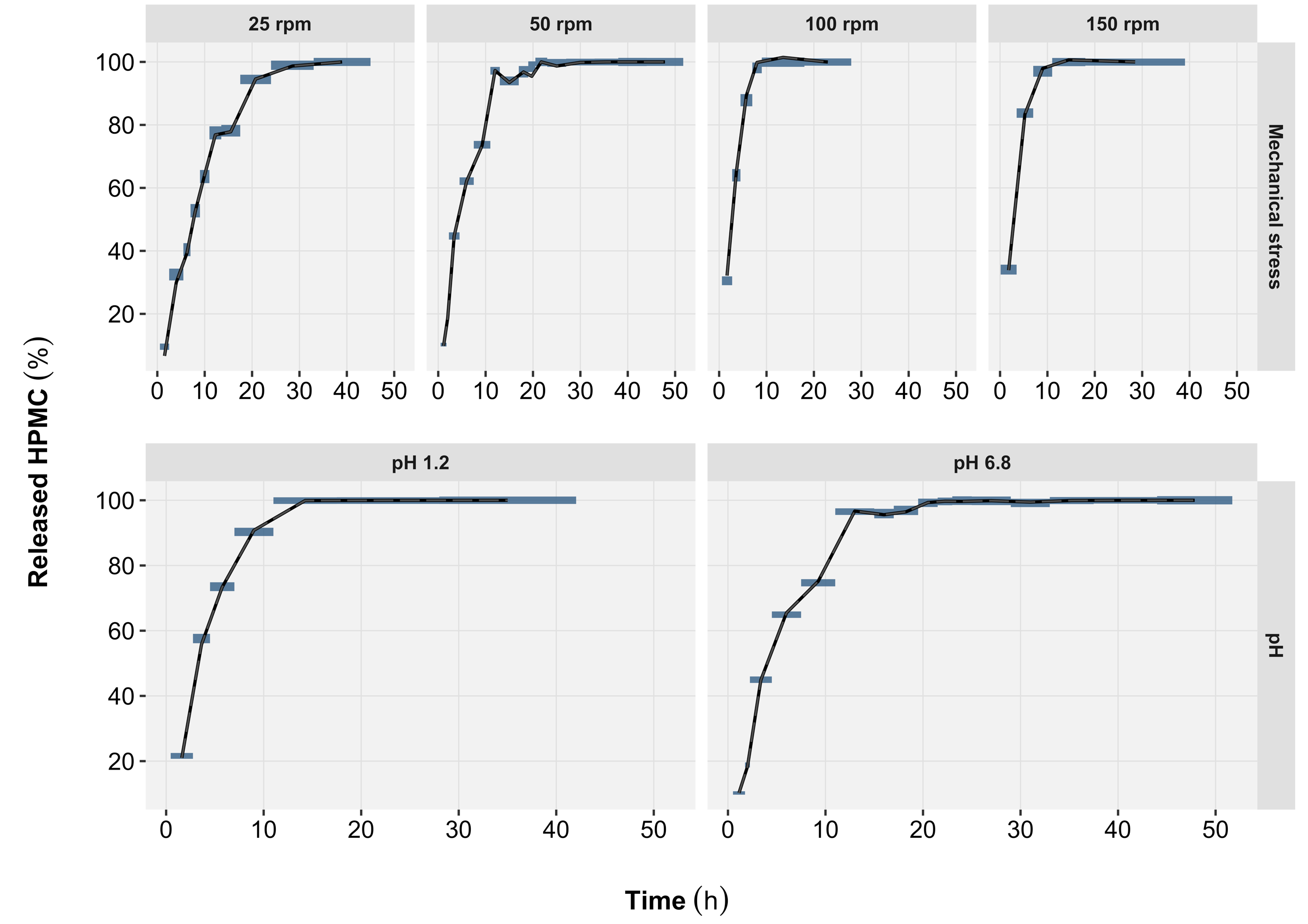
**

**Figure S1a and S1b:** Prediction corrected visual predictive checks (VPC) of *in vitro* released HPMC time course of the formulation (*S1a*) and experimental conditions (*S1b*) related covariates. With prediction corrected VPC the observed and simulated values of the dependent variable (released HPMC) are for each bin normalized to the typical model prediction (i.e. no between tablet variability) at the median of the independent variable (time) in the bin. The median (bold line) of the observed data is compared to the 95% confidence intervals (shaded areas) for the median of the simulated (n = 1000) data.
