# Supplementary material for: In Vitro and In Vivo Modeling of Hydroxypropyl Methylcellulose (HPMC) Matrix Tablet Erosion Under Fasting and Postprandial Status
Source: Pharm Res. 2017 Feb 2;34(4):847–59. doi: 10.1007/s11095-017-2113-7 (PMC5336534; doi:10.1007/s11095-017-2113-7)
Supplement: Supplementary file 2 — (DOCX 46 kb) [file 11095_2017_2113_MOESM2_ESM.docx]

**Figure S2:** NONMEM model code of the *in vivo* tablet erosion model

$SIZES LVR=33 *; Increase NONMEM default number of ETA and EPS*

$PROBLEM In vivo HPMC tablet erosion model

$INPUT **ID** *; Subject identifier (From 1 to 5)*

FORM *; Flag for tablet formulation*

FSTD *; Flag for prandial status*

*; (1: Fasting 0: Postprandial)*

EXC *; Flag for experimental conditions*

*; (formulation + prandial status)*

**TIME** *; Time since the start of the crossover study (h)*

TAD *; Time since the last tablet ingestion (h)*

**DV** *; Dependent variable (Released HPMC)*

TBLWT *; Initial tablet weight (mg)*

**DOSE** *; Amount of HPMC in tablet (mg) [all record]*

**AMT** *; Amount of HPMC in tablet (mg) [dosing records]*

**EVID** *; NONMEM event identifier (0: observation,*

*; 1: dose, 2: dummy rows, 4:dose w/ reset)*

GET *; Gastric emptying time (h)*

CAT *; Colon arrival time (h)*

MK4 *; Fraction of high molecular weight HPMC (%)* MK100 *; Fraction of low molecular weight HPMC (%)* DCP *; Fraction of calcium hydrogen phosphate (%)*

$DATA In_vivo_data.csv

**IGNORE**=@ *; Ignore column headers*

**IGNORE**=(EXC.EQ.5) *; Exclude data form formulation no. 4 under*

*; postprandial status*

$SUBROUTINE **ADVAN13 TOL**=9

$MODEL **COMP**=(TABLET, **DEFDOSE**) *; 1. Tablet compartment*

**COMP**=(MEDIA, **DEFOBS**) *; 2. Media compartment*

$PK

*; Occasions*

| VI1 = | 0 |  | | | | | | | | |
| --- | --- | --- | --- | --- | --- | --- | --- | --- | --- | --- |
| VI2 = | 0 |  |  |  |  |  |  |  |  |  |
| VI3 = | 0 |  |  |  |  |  |  |  |  |  |
| VI4 = | 0 |  |  |  |  |  |  |  |  |  |
| VI5 = | 0 |  |  |  |  |  |  |  |  |  |
| VI6 = | 0 |  |  |  |  |  |  |  |  |  |
| IF(EXC |  | == | 1) | VI1 | = | 1 | *;* | *Formulation* | *1,* | *Fasting* |
| IF(EXC |  | == | 2) | VI2 | = | 1 | *;* | *Formulation* | *2,* | *Fasting* |
| IF(EXC |  | == | 3) | VI3 | = | 1 | *;* | *Formulation* | *3,* | *Fasting* |
| IF(EXC |  | == | 4) | VI4 | = | 1 | *;* | *Formulation* | *3,* | *Postprandial* |
| IF(EXC |  | == | 5) | VI5 | = | 1 | *;* | *Formulation* | *4,* | *Fasting* |
| IF(EXC |  | == | 6) | VI6 | = | 1 | *;* | *Formulation* | *4,* | *Postprandial* |

*; Inter occasion variability (IOV) on pH stomach* IOSFS = VI1***ETA**(1)+VI2***ETA**(2)+VI3***ETA**(3)+VI5***ETA**(4) IOSFD = VI4***ETA**(5)+VI6***ETA**(6)

*; IOV pH proximal SI*

IOJ = VI1***ETA**(7)+VI2***ETA**(8)+VI3***ETA**(9)+VI4***ETA**(10)+VI5***ETA**(11)+VI6***ETA**(12)

*; IOV pH distal SI*

IOI = VI1***ETA**(13)+VI2***ETA**(14)+VI3***ETA**(15)+VI4***ETA**(16)+VI5***ETA**(17)+VI6***ETA**(18)

*; IOV pH colon*

IOC = VI1***ETA**(19)+VI2***ETA**(20)+VI3***ETA**(21)+VI4***ETA**(22)+VI5***ETA**(23)+VI6***ETA**(24)

*; Covariates*

*; Mechanical stress*

S_RPM = **THETA**(1) *; RPM in stomach* J_RPM = **THETA**(2) *; RPM in proximal SI* I_RPM = **THETA**(3) *; RPM in distal SI* C_RPM = **THETA**(4) *; RPM in colon*

*; Gastrointestinal pH*

S_PH = FSTD*1.73*EXP(IOSFS) + (1-FSTD)*4.9*EXP(IOSFD) *; pH in stomach*

J_PH = 6.63 * EXP(IOJ) *; pH in proximal SI* I_PH = 7.49 * EXP(IOI) *; pH in distal SI* C_PH = 6.63 * EXP(IOC) *; pH in colon*

*; Proximal/distal SI transfer*

| JTT | = **THETA**(6) | *;* | *Proximal SI transit time (h)* |
| --- | --- | --- | --- |
| IAT | = GET + JTT | *;* | *Arrival time in distal SI (h)* |

*; Structural model parameters*

IO_VM = VI1***ETA**(26)+VI2***ETA**(27)+VI3***ETA**(28)+VI4***ETA**(29)+VI5***ETA**(30)+VI6***ETA**(31)

VM_VAR = 1 * EXP(**ETA**(25)+IO_VM) *; Total variability Maximal HPMC release rate*

**ALAG1** = **THETA**(5) *; Lag time (h)*

$DES

*; Compartment Amounts*

X1 = **A**(1) *; Amount of HPMC left in tablet (mg)*

X2 = **A**(2) *; Amount of HPMC released in media (mg)*

IF(X1.LE.0) X1 = 0 *; Code safety to avoid negative amounts*

*; Compute gastrointestinal tablet location*

LOCA = 0

IF(**T**.LT.GET) LOCA = 1 *; tablet in stomach* IF(**T**.GE.GET.**AND**.**T**.LT.IAT) LOCA = 2 *; tablet in proximal SI* IF(**T**.GE.IAT.**AND**.**T**.LT.CAT) LOCA = 3 *; tablet in distal SI* IF(**T**.GE.CAT) LOCA = 4 *; tablet in colon*

*; Adjust mechanical stress based on tablet location*

RPM = 0 *; Initialize RPM*

IF(LOCA.EQ.1) RPM = S_RPM *; Set to RPM in stomach* IF(LOCA.EQ.2) RPM = J_RPM *; Set to RPM in proximal SI* IF(LOCA.EQ.3) RPM = I_RPM *; Set to RPM in distal SI* IF(LOCA.EQ.4) RPM = C_RPM *; Set to RMP in colon*

*; Adjust pH based on tablet location*

PH = 0 *; Initialize pH* IF(LOCA.EQ.1) PH = S_PH *; Set to pH in stomach* IF(LOCA.EQ.2) PH = J_PH *; Set to pH in proximal SI* IF(LOCA.EQ.3) PH = I_PH *; Set to pH in distal SI* IF(LOCA.EQ.4) PH = C_PH *; Set to pH in colon*

*; Covariates on maximal HPMC release rate (Vmax)*

ERPMVX = (1 + 0.0115114 * (RPM - 50.00))*; Relative effect of RPM on Vmax*

EPHVX = (1 - 0.0318760 * (PH - 6.80)) *; Relative effect of pH on Vmax*

EMK4VX = (1 - MK4 / (18.7153 + MK4)) *; Relative effect of MK4 on Vmax*

EDCPVX = (1 + 0.0331567 * (DCP - 57.60))*; Relative effect of DCP on Vmax*

COVVX = EDCPVX * EMK4VX * EPHVX * ERPMVX*; Relative effect of all COV on Vmax*

*; Covariates on the amount of HPMC in tablet at which the release rate*

*; is half of Vmax (Km)*

EPHKM = (1 + 0.0934523 * (PH - 6.80)) *; Relative effect of pH on Km*

EDCPKM = (1 + 0.0521938 * (DCP - 57.60))*; Relative effect of DCP on Km*

KMCOV = EDCPKM * EPHKM *; Relative effect of all covariates on Km*

*; Structural model parameters*

TVVX = 21.9546 * COVVX *; Typical value of Vmax*

VMAX = TVVX * VM_VAR *; Individual value of Vmax*

**KM** = 34.1559 * COVKM *; Typical value of Km*

*; Erosion Model*

*; Release rate equation*

ER = (VMAX * X1) / (**KM** + X1) *; HPMC release rate (mg/h)*

*; Differential equations*

**DADT**(1) = -ER *; Amount of HPMC left in tablet (mg)*

**DADT**(2) = ER *; Amount of HPMC released in media (mg)*

$ERROR

A1 = **A**(1) *; Amount of HPMC left in tablet (mg)*

A2 = **A**(2) *; Amount of HPMC released in media (mg)*

IF(A1.LE.0) A1 = 0 *; Code safety to avoid negative amounts*

*; Compute released HPMC (%)*

NPDIS = (A2 / **DOSE**) * 100 *; Normalized percent of HPMC dissolved*

IF(NPDIS.LE.1E-10) NPDIS = 1E-10 *; Code safety to avoid negative amounts*

**IPRED** = NPDIS *; Individual released HPMC predictions*

**Y** = **IPRED** * (1+**EPS**(1)) + **EPS**(2)*; Released HPMC fit*

W = SQRT((**IPRED***SQRT(SIGMA(1,1)))**2 + SIGMA(2,2)) *; Residuals weight*

IRES = **DV**-**IPRED** *; Individual residuals*

IWRES = 0

IF(W.GT.0) IWRES = IRES/W *; Individual weighted residuals*

$THETA

(0,42.0432505061042) *; Th1. rpm stomach (rpm)* (0,97.7755289502576) *; Th2. rpm duodenum/proximal SI (rpm)* (0,28.6148105175676) *; Th3. rpm distal SI (rpm)* (0,10.7024328122356) *; Th4. rpm colon (rpm)* (0,0.347727058143364) *; Th5. lag time (h)*

1 **FIX** *; Th6. transfer time between prox./distal* *SI (h)*

*; Variances for pH have been calculated as (SD/MEAN)**2 from literature values*

*; for details refer to Table III of the manuscript.*

$OMEGA **BLOCK**(1) 0.090 **FIX** *; Om1.1. BOV pH stomach fasting (Visit 1)*

$OMEGA **BLOCK**(1) **SAME** *; Om2.2. BOV pH stomach fasting (Visit 2)*

$OMEGA **BLOCK**(1) **SAME** *; Om3.3. BOV pH stomach fasting (Visit 3)*

$OMEGA **BLOCK**(1) **SAME** *; Om4.4. BOV pH stomach fasting (Visit 5)*

$OMEGA **BLOCK**(1) 0.027 **FIX** *; Om5.5. BOV pH stomach postprandial (Visit 4)*

$OMEGA **BLOCK**(1) **SAME** *; Om6.6. BOV pH stomach postprandial (Visit 6)*

$OMEGA **BLOCK**(1) 0.0064 **FIX** *; Om7.7. BOV pH proximal SI (Visit 1)*

$OMEGA **BLOCK**(1) **SAME** *; Om8.8. BOV pH proximal SI (Visit 2)*

$OMEGA **BLOCK**(1) **SAME** *; Om9.9. BOV pH proximal SI (Visit 3)*

$OMEGA **BLOCK**(1) **SAME** *; Om10.10. BOV pH proximal SI (Visit 4)*

$OMEGA **BLOCK**(1) **SAME** *; Om11.11. BOV pH proximal SI (Visit 5)*

$OMEGA **BLOCK**(1) **SAME** *; Om12.12. BOV pH proximal SI (Visit 6)*

$OMEGA **BLOCK**(1) 0.0038 **FIX** *; Om13.13. BOV pH distal SI (Visit 1)*

$OMEGA **BLOCK**(1) **SAME** *; Om14.14. BOV pH distal SI (Visit 2)*

$OMEGA **BLOCK**(1) **SAME** *; Om15.15. BOV pH distal SI (Visit 3)*

$OMEGA **BLOCK**(1) **SAME** *; Om16.16. BOV pH distal SI (Visit 4)*

$OMEGA **BLOCK**(1) **SAME** *; Om17.17. BOV pH distal SI (Visit 5)*

$OMEGA **BLOCK**(1) **SAME** *; Om18.18. BOV pH distal SI (Visit 6)*

$OMEGA **BLOCK**(1) 0.010 **FIX** *; Om19.19. BOV pH colon (Visit 1)*

$OMEGA **BLOCK**(1) **SAME** *; Om20.20. BOV pH colon (Visit 2)*

$OMEGA **BLOCK**(1) **SAME** *; Om21.21. BOV pH colon (Visit 3)*

$OMEGA **BLOCK**(1) **SAME** *; Om22.22. BOV pH colon (Visit 4)*

$OMEGA **BLOCK**(1) **SAME** *; Om23.23. BOV pH colon (Visit 5)*

$OMEGA **BLOCK**(1) **SAME** *; Om24.24. BOV pH colon (Visit 6)*

$OMEGA 0.0227867597125082 *; Om25.25. BSV Vmax*

$OMEGA **BLOCK**(1) 0.0252260860575006*; Om26.26. BOV Vmax (Visit 1)*

| $OMEGA | **BLOCK**(1) | **SAME** | *;* | *Om27.27.* | *BOV* | *Vmax* | *(Visit* | *2)* |
| --- | --- | --- | --- | --- | --- | --- | --- | --- |
| $OMEGA | **BLOCK**(1) | **SAME** | *;* | *Om28.28.* | *BOV* | *Vmax* | *(Visit* | *3)* |
| $OMEGA | **BLOCK**(1) | **SAME** | *;* | *Om29.29.* | *BOV* | *Vmax* | *(Visit* | *4)* |
| $OMEGA | **BLOCK**(1) | **SAME** | *;* | *Om30.30.* | *BOV* | *Vmax* | *(Visit* | *5)* |
| $OMEGA | **BLOCK**(1) | **SAME** | *;* | *Om31.31.* | *BOV* | *Vmax* | *(Visit* | *6)* |

$SIGMA

0.0213317947870998 *; Sig1.1. Proportional Error*

3.03520322846441 *; Sig2.2. Additive Error*

*; Parameter estimation settings*

$ESTIMATION **METHOD**=1 **SIGL**=9 **NSIG**=3 **INTER MAXEVAL**=9999 **PRINT**=1 **NOABORT**

*; Compute parameters uncertainty*

$COVARIANCE **UNCONDITIONAL**

*; Generate output table*

$TABLE **ID TIME** TAD **EVID DOSE DV IPRED** A1 A2 **ETAS**(1:**LAST**) IWRES **CWRES** VMAX **KM** FORM FSTD EXC TBLWT GET CAT JTT IAT PH RPM LOCA MK4 MK100 DCP **NOPRINT ONEHEADER FILE**=sdtab001
